# Microsaccade Rate Varies with Subjective Visibility during Motion-Induced Blindness

**DOI:** 10.1371/journal.pone.0005163

**Published:** 2009-04-09

**Authors:** Po-Jang Hsieh, Peter U. Tse

**Affiliations:** Department of Psychological and Brain Sciences, Moore Hall, Dartmouth College, Hanover, New Hampshire, United States of America; University of Minnesota, United States of America

## Abstract

Motion-induced blindness (MIB) occurs when a dot embedded in a motion field subjectively vanishes. Here we report the first psychophysical data concerning effects of microsaccade/eyeblink rate upon perceptual switches during MIB. We find that the rate of microsaccades/eyeblink rises before and after perceptual transitions from not seeing to seeing the dot, and decreases before perceptual transitions from seeing it to not seeing it. In addition, event-related fMRI data reveal that, when a dot subjectively reappears during MIB, the blood oxygen-level dependent (BOLD) signal increases in V1v and V2v and decreases in contralateral hMT+. These BOLD signal changes observed upon perceptual state changes in MIB could be driven by the change of perceptual states and/or a confounding factor, such as the microsaccade/eyeblink rate.

## Introduction

When high-contrast stationary stimuli, such as dots, are embedded within a global moving pattern, such as a rotating field of crosses, the dots seem to disappear and reappear alternately, although they are in fact constantly present in the stimulus [1]. At least two hypotheses have been raised to account for the phenomenon of motion-induced blindness (MIB). The first hypothesis argues that MIB is related to surface completion [2] or perceptual filling-in [3]. Perceptual filling-in is also known as ‘perceptual fading’ or ‘The Troxler effect’ [4], which occurs when an object, though present in the world and continually casting light upon the retina, vanishes from visual consciousness to be replaced by its surrounding background. This phenomenon is commonly thought to arise because of bottom-up local sensory adaptation to edge information [5] and is presumed to occur early in the visual pathway, such as in the lateral geniculate nucleus of the thalamus (LGN) or even retinal ganglion cells [6]–[8]. Because perceptual fading involves both loss of signal about the presence of an object, and filling in of the background in place of the object, it is possible that the effect has both a retinal and a cortical component arising from neuronal adaptation and filling-in, respectively. Retinal and cortical accounts are not mutually exclusive. For example, retinal adaptation could lead to a weakened edge signal sent from retinal ganglion cells, followed by a cortical filling-in process [9], [10].

A second hypothesis maintains that MIB may be a kind of perceptual rivalry [11] between the motion background and the target, perhaps arising because of attentional mechanisms [1]. Proponents of this view argue that MIB is unlikely to reflect retinal suppression, sensory masking or adaptation [1], [12], [13]. They point out that if there are several stationary dots present in a MIB display, sometimes one dot will disappear while the others may not [1]. Similarly, if several dots have disappeared, only one might reappear, while the others will remain unseen. If retinal stabilization were the sole cause of MIB, fixation would stabilize all dots equally, and all would disappear together. Conversely, the fact that dot reappearance can also be piecemeal suggests that the mechanism underlying reappearance is not simply due to breaks in retinal stabilization induced by blinks or microsaccades. Such breaks would again occur over the entire retinal image, and thus should lead to reappearance of all dots together. It has also been shown that object and grouping procedures influence MIB [1], [13]. For example, when two triangles are overlapping (i.e., making a star of David pattern with one triangle green and the other red) two legs of one triangle are more likely to go into and out of MIB together than two legs from different triangles [1]. Furthermore, changes to an object's characteristics while that object has vanished under MIB, can affect how the information returns to awareness [13]. The finding that MIB can continue even during movement of perceptually vanished objects also suggests that MIB is not caused by bottom-up local sensory adaptation [1], [13].

Here we examine both the microsaccade rate and eyeblink rate upon perceptual switches during MIB. If MIB involves bottom-up local sensory adaptation to edge information, we would expect to see a change in microsaccade/eyeblink rate before a target reappears from motion-induced blindness, similar to what has been observed during Troxler fading [14]. On the contrary, if bottom-up local sensory adaptation to edge information is not necessary to induce MIB, we would expect to see no change in microsaccade/eyeblink rate before a target reappears from motion-induced blindness.

In a second experiment, we apply event-related functional magnetic resonance imaging (fMRI) to examine the neural basis of subjective visibility following the induction of MIB.

## Results

**Figure 1:**
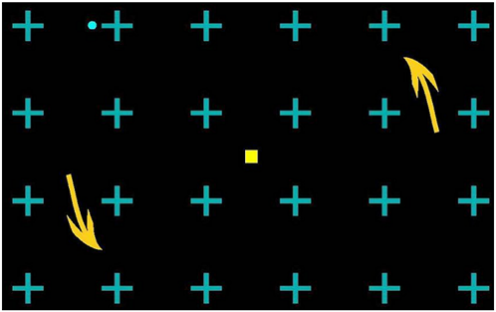
Stimuli. The rotating background stimulus for inducing MIB. It consisted of cyan crosses on a black background rotating counterclockwise at 70°/sec, as indicated by the yellow arrows (not present in stimulus). After fixating on the central fixation spot for several seconds, the cyan dot disappears and reappears alternatively.

**Figure 2:**
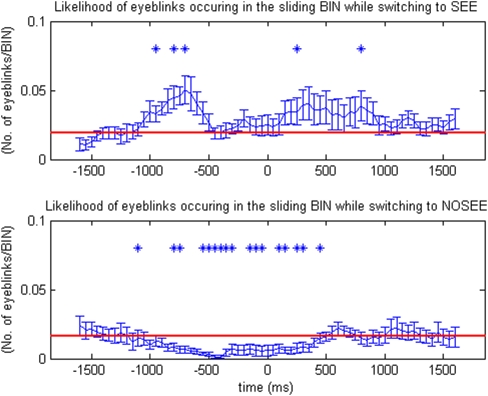
Rates of eyeblinks (n = 10) around the time of a perceptual switch (time 0). The rate of eyeblinks rises before and after perceptual transitions from ‘no see’ to ‘see’, and decreases during perceptual transitions from ‘see’ to ‘no see.’ In the two-tailed simple t-test, those data points that are significantly different than the baseline (red line) are marked as * (p<0.05)

**Figure 3:**
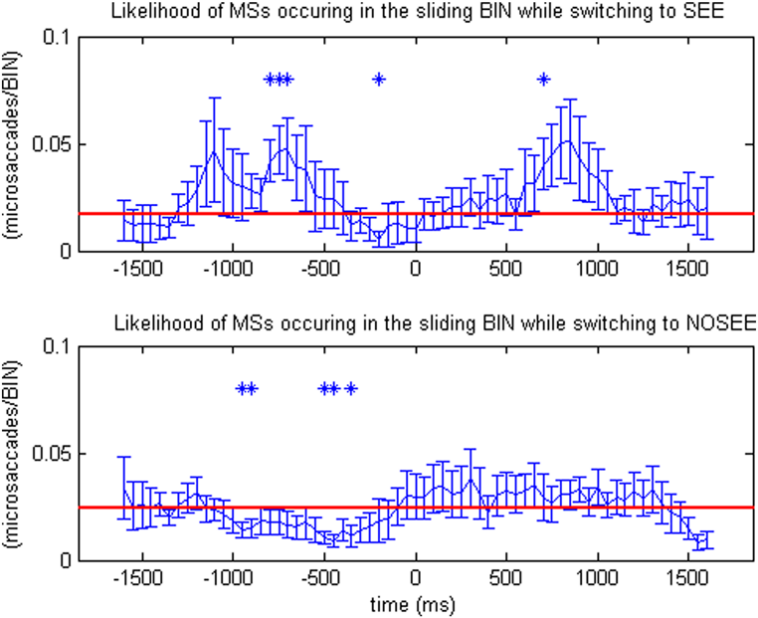
Rates of microsaccades around the time of a perceptual switch (time 0). The rate of microsaccades rises before and after perceptual transitions from ‘no see’ to ‘see’, and decreases before perceptual transitions from ‘see’ to ‘no see.’ In the two-tailed simple t-test, those data points that are significantly different than the baseline (red line) are marked as * (p<0.05).

In each of the four stimulation blocks, a single target dot was presented in one of the four quadrants (left top, left bottom, right top, or right bottom) on a motion background (**Figure 1**). Subjects were required to indicate their perceptual state by pressing a button. **Figures 2** and **3** show that the microsaccade/eyeblink rate was correlated with the type of perceptual switches during MIB. In **Figure 2**, eyeblink rate was significantly greater than the baseline rate both before and after a perceptual switch to the ‘see’ condition. In contrast, eyeblink rate was significantly smaller than the baseline both before and after a perceptual switch to the ‘no see’ condition. **Figure 3** shows that the microsaccade rate was significantly greater than baseline both before and after a perceptual switch to the ‘see’ condition, and was significantly smaller than the baseline only before a perceptual switch to the ‘no see’ condition.

In the fMRI experiment, we further examine the neural basis of subjective visibility following the induction of MIB. We compare the BOLD signal after perceptual switches by averaging data from ‘see’ conditions that followed ‘no see’ conditions, and vice versa. Since receptive fields within a given retinotopic ‘quadrant’ (left hemisphere V1v for example) only receive bottom-up input from one quadrant of the visual field (e.g. right top), we can compare the BOLD signal in different conditions to see whether the BOLD signal only changes when the target dot undergoing MIB is directly located in the corresponding quadrant.

**Figure 4:**
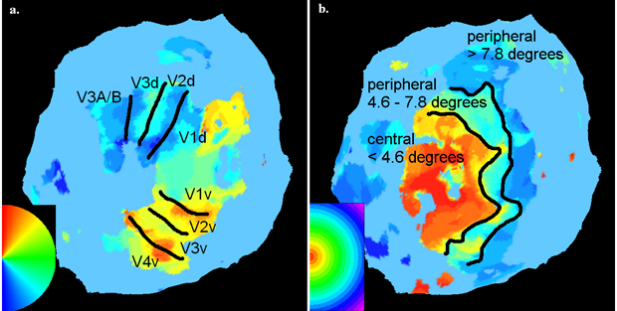
Retinotopy. (a) A typical retinotopic map of the flattened left hemisphere occipital pole for one subject is shown with the approximate borders between the retinotopic areas specified in black. Retinotopic area masks were individually specified for each hemisphere of each subject. Blue here represents the lower vertical meridian, cyan/green the horizontal meridian, and red the vertical meridian. (b) A typical retinotopic map of the flattened left hemisphere occipital pole for one subject is shown with the approximate borders specified in black between the central (<4.6 visual degrees), middle (4.6–7.8 visual degrees), and peripheral (>7.8 visual degrees) areas.

Regions of interest (ROIs) in the current study include individually specified retinotopic areas V1v, V1d, V2v, V2d, V3v, V3d, V3A/B, and V4v (**Figures 4**). We focus on these visual areas because it has been shown that neuronal activity in retinotopic visual areas is correlated with perceptual rivalry and attentional modulation [15]. Examining BOLD responses in these areas during MIB should help us to understand whether these phenomena are related. In addition to these areas, localizer scans were performed to isolate hMT+, which is of particular interest in this study because hMT+ is an area of the brain known to process motion [16]–[20].

**Figure 5:**
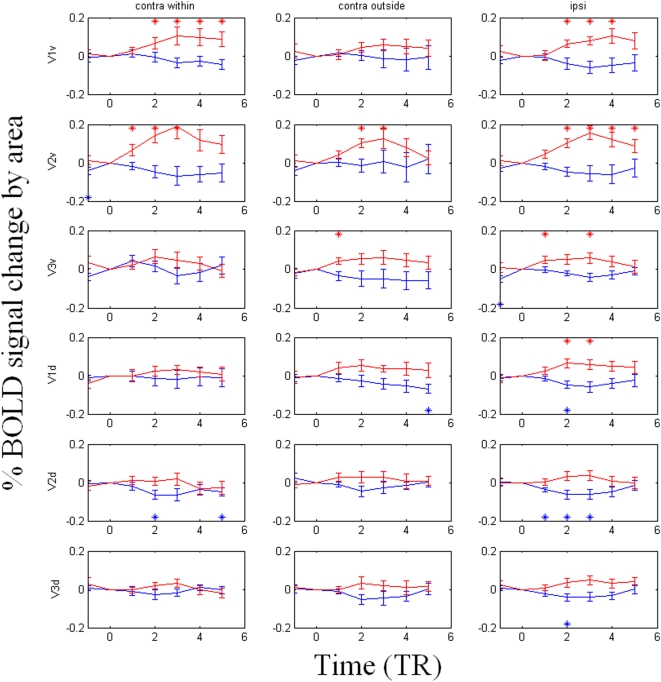
The differences of BOLD timecourses (TR = 1.6 seconds) upon perceptual switches in V1v, V1d, V2v, V2d, V3v, and V3d. The BOLD signal change averaged across voxels within subjects' ROIs and across hemispheres relative to the 16 slice volume acquisition (TR) = 0 position, corresponding to the beginning of a volume in which the subject reported a perceptual switch. The area is marked ‘contra within’ when the target was located inside the corresponding visual field, and marked ‘contra outside’ when target was located on the contralateral side to the ROI but outside the corresponding visual field. The area is marked ‘ipsi’ when the ROI was on the same side as the target that underwent MIB. The x-axis shows the time in units of TR (1.6 seconds), and the y-axis shows the percentage change of BOLD signal (%). The results show that the BOLD signal increased when the stimulus reappeared from MIB in V1v and V2v. The same result was observed when the stimulus was presented ipsilaterally to these areas. Statistics for Figures 5 and 6: N = 14; A two-tailed t-test was carried out to compare the value of TR = 0 (set to be zero) to the means of each TR individually. Those data points that are significantly different than 0 are marked as ‘*’ (p<0.05).

**Figure 6:**
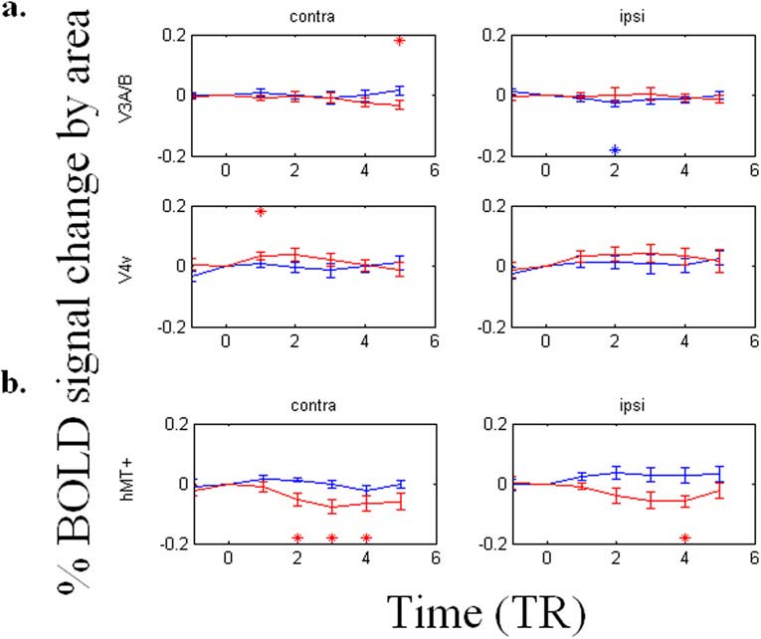
The differences of BOLD signal timecourses (TR = 1.6 seconds) upon perceptual switches in V3A/B, V4v, and hMT+. (a) The BOLD signal modulates weakly in V3A/B or V4v. (b) The BOLD signal modulates with the percept in contralateral hMT+, in a manner largely opposite that of V1v and V2v. In particular, BOLD signal decreases upon subjective disappearance of the dot in hMT+, but increases in V1 and V2.

**Figure 5** shows the average timecourse data for the ‘see’ and ‘no see’ states. An asterisk above or below a data point, in the color of that data point indicates that it differs significantly (p<0.05) from TR_0_ (the BOLD signal at the time of a perceptual state change set to zero). Our results show that, in general, early visual areas have a stronger BOLD activation after a perceptual switch (**Figure 5**) such that signal intensity rises after perceptual transitions from ‘no see’ to ‘see’ (pink) for the case where the dot was in fact present in the corresponding contralateral quadrant. Surprisingly, BOLD signal deviations reached significance not only when the target was located within these ROI's contralateral ‘within’ quadrant (as opposed to the contralateral quadrant where the dot was not present), which might have been expected, but also when the target was located ipsilaterally to these ROIs, which was far from expected. BOLD signal modulates relatively more weakly with perceptual state in contralateral or ipsilateral V3v, V3d (**Figure 5**), V3A or V4v (**Figure 6a**). In area hMT+ (**Figure 6b**), the BOLD signal ***decreases*** upon transition to ‘see’ when the target dot was presented contralaterally, which is opposite the BOLD response pattern evident in areas V1v and V2v.

## Discussion

An increase in microsaccade rate before target reappearance from perceptual fading was recently reported [14]. This finding confirms that microsaccades or blinks likely break the bottom-up local sensory adaptation to edge information that is presumably necessary for perceptual fading to occur. Our results are consistent with these findings, showing that the microsaccade rate increases before perceptual switches to the ‘see’ condition during MIB. This finding suggests that bottom-up local sensory adaptation to edge information may play some role during MIB, and is in line with other evidence that there are some similarities between MIB and Troxler fading [2], [3].

An interesting finding is that the microsaccade/eyeblink rate generally increases after perceptual switches to the ‘see’ states, and decreases after perceptual switches to the ‘no see’ state. One possible explanation is that shifts in attention may alter the baseline microsaccade rate. Several authors have reported changes in the baseline rate of microsaccades after the onset of a peripheral cue that captures attention in humans [21], [22], [23] and monkeys [24]. The decrease and then increase of microsaccade rate might simply be the result of subjects paying either more or less attention immediately before or immediately after the change in percept. It is possible that when the target is not perceived, subjects paid more or less attention to the stimuli and therefore made fewer microsaccades.

Our fMRI data show that, among visual areas tested, the BOLD response increases when the target dot, although continually present in the stimulus, subjectively reappears following MIB in V1v and V2v. In contrast, the BOLD response decreases when the target reappears following MIB in hMT+. Interestingly, modulation of the BOLD signal occurred in corresponding ipsilateral areas as well, although the physical target was only located in one quadrant of the visual field. This result is consistent with the possibility that eyeblinks and/or fixational eye movements, which are correlated with switches in perceptual state, may lead to changes in neuronal firing rate in V1 [25]–[27] and ipsilateral and bilateral BOLD signal changes [28].

Because we cannot rule out this possible confound of microsaccades, it might seem that the present dataset should be recollected with an fMRI eyetracker that has a high enough temporal and spatial resolution to permit detection of microsaccades. This, however, would not settle the matter, and it is difficult to imagine a method for presenting MIB that can get around this problem, short of image stabilization upon the retina, which might itself eliminate MIB entirely. First, at present, to our knowledge, no commercially available eyetracker can detect microsaccades reliably in the scanner. Second, even if microsaccades could easily be detected in the scanner, the eye movements measured in the scanner would presumably demonstrate what we demonstrated here psychophysically by measuring eye movements during MIB outside of the scanner; Namely, measuring eye movements in the scanner will presumably also show that the probablility of microsaccade or eyeblink occurrence changes prior to and/or subsequent to the onset of MIB. Thus the potential confound will remain even if eye movements are measured in the scanner. Nonetheless, one recent study [29] has managed to detect the neural correlates of microsaccades in a few subjects. They found that BOLD signal increases in V1 and V2, but remains unchanged in hMT+ bilaterally following a microsaccade, and increases in all these areas following an eyeblink. Thus our data in V1 or V2 could arise from microsaccades or eyeblinks, but this is not the case for hMT+, where we see a decrease in activity following motion-induced blindness. Thus, despite the possibility of confounding eye movement effects in V1 and V2, although not for hMT+, it is useful to publicize the present results as a first step toward determining the neural correlates of visibility in motion-induced blindness.

If the bilateral BOLD activation we observe is not solely due to confounding fixational eye movements that tend to occur at times of perceptual transition between ‘see’ and ‘no see’ states, what other possible factors might be involved? One possible model that could account for the present data is one according to which the bilateral BOLD signal modulation is caused by a corticocortical or corticothalamocortical feedback mechanism from contralateral to ipsilateral cortex. It is possible that each perceptual switch in MIB is necessarily accompanied by an attentional change, which could lead to a BOLD signal change through feedback activation [30]–[39]. Another possibility is that this feedback activation, if any, could be directly related to visual awareness [15], [40]–[43] (but see [44], [45]). Other explanations of the data include retinal adaptation, or possibly amplification of retinal adaptation effects at a cortical level, due to some suppressive effects of the surround (e.g., surround suppression) on the target. This need not necessarily involve feedback, and could reflect a feedforward mechanism.

Why does the BOLD response follow an opposite timecourse upon perceptual switches during MIB in hMT+ and V1v? During MIB, the dot appears to vanish, and to be replaced by the moving background pattern. Even if no actual motion ever traverses the location of the cyan spot, it seems that a moving surface does traverse the location of the cyan dot once it has vanished from awareness. This could comprise an instance of perceptual filling-in. The location of the vanished cyan dot appears to be filled in with motion in the sense that the crosses appear to lie on a rotating black surface [46]. If more motion is perceived when the dot has been filled in, hMT+ might respond more in the ‘no see’ case. Another possibility is that the percept of the target cyan dot and the percept of the background crosses might compete with each other. In the ‘see’ case, less motion is perceived because the target cyan dot is seen; in the ‘no see’ case, more motion is perceived because the target cyan dot is not seen. These possibilities might explain our finding that the BOLD signal in hMT+ falls after transitions from ‘no see’ to ‘see’. As for the BOLD signal change upon the stimulus onset/offset in hMT+, it is possible that the BOLD signal goes up after physical target onset because neurons in this area become more active upon stimulus onset.

To conclude, we report the first psychophysical data concerning effects of microsaccade/eyeblink rate upon perceptual switches during MIB. We find that the rate of microsaccades/eyeblink rises before and after perceptual transitions from not seeing to seeing the dot, and decreases before perceptual transitions from seeing it to not seeing it. This finding suggests that bottom-up local sensory adaptation to edge information may play some role for the induction of MIB. We also report event-related fMRI data indicating brain areas where BOLD signal is correlated with subjective visibility of a target following the induction of motion-induced blindness. We find BOLD signal modulation to be correlated with conscious visibility of the target in bilateral V1, V2, and hMT+. The correlation with perceptual state may arise because the neuronal correlates of perceptual state exist in the ROIs where these correlations are observed. Or they may arise because of confounding fixational eye movements that may tend to occur at points of perceptual transition between ‘see’ and ‘no see’ states, attentional effects, or other forms of feedback, in which case BOLD signal correlates of perceptual state during MIB would not be indicative of the direct neural basis of these perceptual states. Future experiments are required to unambiguously specify the exact mechanism underlying correlations between BOLD signal changes and changes in perceptual state observed during MIB. While the present data do not unambiguously specify the neural correlates underlying visibility in motion-induced blindness, they do place useful constraints on what and where those neural correlates might be. In particular, our finding that BOLD signal drops bilaterally in hMT+ upon induction of motion-induced blindness, cannot be attributed to microsaccades or eyeblinks, because these have recently been found to not induce a decrease in BOLD signal in hMT+ following their occurrence [29].

## Materials and Methods

### Psychophysics methods and procedures

Ten healthy volunteers (seven paid Dartmouth students and the authors) participated in the experiment. Each run lasted 307.2 sec and contained 4 stimulation blocks (76.8 sec each). In each stimulation block, a cyan (CIE x = 0.222, y = 0.331) dot was presented in one of the four quadrants (left top, left bottom, right top, and right bottom) on a black (CIE x = 0.275, y = 0.437) background on which cyan (CIE x = 0.204, y = 0.272) cross elements arranged in a grid rotated counterclockwise around the center point at a constant speed (70°/sec) (**Figure 1** **)**. The order of the 4 stimulation blocks, each containing a cyan dot in one of the four quadrants, was randomized without replacement for each run. The background contained cyan crosses and rotated continuously. Each cyan cross subtended 0.75° in height and 0.75° in width, and was centered 2.25° from other cyan crosses horizontally and vertically. The bars comprising the cross subtended 0.75° in height and 0.12° in width. The cyan dot had a diameter subtending 0.25° visual angle, centered 4° (left or right from vertical midline) and 3° (above or below horizontal midline) depending on which quadrant it was located in. The contour of the cyan dot was blurred by equally mixing the color of the dot with the color of the black background. This consisted of the equiluminant cyan center, a dot of diameter 0.08°, surrounded by a linear gradient between cyan and black that brought the overall blurred dot diameter to 0.12° visual angle. Subjects reported their current perceptual state by pressing a button with their right hand. They were asked to press the right-hand button when they did not see the cyan dot even if they knew it to be there, and release the button when they did see it.

Eye movements were recorded using a SRresearch Eyelink2 system for the left eye. Eye position was sampled at 250Hz. Observers ran in one or two sessions, each equivalent to a run of the fMRI experiment. Observers were required to maintain fixation on each trial. A miniature video camera, attached to an adjustable headband and bar, was fitted about 2 cm below the subject's left eye, and eye movements were calibrated to a dot that moved to nine positions on the screen in random order. Observers rested their chin in a stable rest. The distance from their eyes to the screen was adjusted such that the visual angles of the stimuli were the same as that in the scanner. The head was not otherwise constrained, although observers were instructed to maintain their head perfectly still. Small head movements could be discounted online by the eye tracker software using the output of four cameras mounted on the monitor. The visual stimulator was a 2 GHz Dell workstation running Windows 2000. The stimuli were presented on a 23-in. SONY CRT gamma-corrected monitor with 1600× 1200 pixels resolution and 85 Hz frame rate.

### Psychophysics Data analysis

Linear drifting in eye traces in both x-channel and y-channel due to headband sliding was corrected before data analysis. We took the total amount of drifting within a run and divided that amount by the total duration of a run. This ‘drifting per unit of time’ was then corrected for each time point. Eyeblinks and huge eye movements were then identified by the algorithm of Engbert and Kliegl [21] (velocity threshold = 10 std.; minimum duration = 5 units of time; velocity type = 2; amplitude bigger than 3.33 visual degrees). Data in a time window starting 400 msec before and ending 600 msec after each eyeblink or huge eye movement were not used in the following analysis. Microsaccades were then located using the same algorithm [21] (velocity threshold = 10 std.; minimum duration = 4 units of time; velocity type = 2; Note that we used a higher velocity threshold than what was used in Engbert and Kliegl [21] to identify eyeblinks and large eye movements. After removing these eyeblinks and large eye movements, we applied the same algorithm again to identify microsaccades). A secondary screening procedure was used to exclude those detected microsaccades that were smaller than 0.15 visual degrees and larger than 2 visual degrees. If there were any two microsaccades (or a cluster of microsaccades) identified by the above algorithm that had an interval shorter than 80ms between them, only the one with the largest amplitude contributed to the final analysis. This was done because microsaccades are followed by a refractory period during which microsaccades do not occur [47].

Each subject's button press time points were identified as the onset of the perceptual switches, which correspond to the 0 points in **Figures 2** and **3**. Within each subject, microsaccade/eyeblink ‘rate’ was plotted by calculating the total number of microsaccade/eyeblink within a 50ms window around each data point. The interval between each data point is 50ms. The value of each data point was then recalculated by taking the mean of the five data points around it. Error bars indicate standard error of the mean rate across subjects. A two-tailed paired t-test was carried out to test whether there was a difference in microsaccade/eyeblink rate from the baseline. Those data points that are significantly different than the baseline are marked as * (p<0.05). The baseline (marked as red lines in **Figures 2** and **3**) is defined by the mean of the first ten data points (−1600ms to −1150ms before the 0 point) and last ten data points (from 1150ms to 1600ms after the 0 point).

### fMRI Experimental design

14 healthy right-handed volunteers with normal depth perception and normal or corrected-to-normal visual acuity (seven females and seven males between the ages of 18 and 41) were run in the experiment. All gave written consent within a protocol passed by the Dartmouth committee for the Protection of Human Subjects and Dartmouth's internal review board. Subjects were paid twenty dollars per session.

Each subject was exposed on average to 6.6±0.3 runs in the scanner (range = 4 to 8). The stimuli properties were identical to the psychophysical setup except that each run lasted 403.2 sec (252 TRs), and in each run, there were 4 stimulation blocks (76.8 sec each), interleaved with 5 ‘no dot’ periods (19.2 sec each). Care was taken to assure that subjects' heads aligned with the vertical meridian of the stimulus. Stimuli were projected from a digital data projector (refresh rate 60Hz) onto a plexiglass screen outside the bore of the magnet, and viewed via a tangent mirror inside the magnet that permitted a maximum of 22°×16° visible area. The projected image was smaller than this and subtended approximately 17°×12°.

### Fixational task

Eye movements, wakefulness, and attention to the fixation point were controlled for during the fMRI experiment by requiring subjects to report whether the fixation had changed color by pressing another button with their left hand. The fixation point was 0.2°×0.2°, located at the center of the screen. The fixation point changed color randomly from blue/yellow to red/green on average every 3.2 seconds. The color change occurred an equal number of times during each block and the same number of times in each run. No motor areas were found to be activated differentially between conditions, corroborating that the motor task was equivalent across all conditions. This task (pressing the lefthand button transiently whenever the fixation point changed color) was demanding and difficult to perform in the absence of fixation. The data show that the average button-press accuracy after a fixation point color change within runs that were included in the analysis was 78.73±2.57% and the average reaction time (RT) was 848.04±53.47 ms. When analyzing color changes of the fixation point as events, the results show that our fixation task does not affect the BOLD signal in the retinotopic areas tested (data not shown). In addition, when analyzing motor responses to the color changes of the fixation point as events, the results also show no BOLD signal modulation in the retinotopic areas tested (data not shown).

### MRI scans

Anatomical and functional whole-brain imaging was performed on a 1.5 T GE Signa scanner. T1-weighted anatomical images were acquired using a high-resolution 3-D spoiled gradient recovery sequence (SPGR; 124 sagittal slices, TE = 6 ms, TR = 16 ms, flip angle = 25°, 1×1×1.2 mm voxels). Functional images were collected using a gradient spin-echo, echo-planar sequence sensitive to BOLD contrast (T2*) (16 slices per volume, 3.75 mm in-plane resolution, 4.5 mm thickness, 1-mm skip, TR = 1600 ms, T2* evolution time = 35 ms, flip angle = 90°).

### fMRI Data Analysis

Data were analyzed offline using BRAIN VOYAGER (BV) 4.9.6 and MATLAB software developed in house. Effects of small head movements were removed using BV's motion correction algorithms. Functional data were not smoothed in the space domain. Low frequency oscillations in the timecourse with periods greater than or equal to 84 TRs (3 cycles per run) were removed.

### Retinotopic mapping

Retinotopy was carried out on all (n = 14) subjects run in the main experiment using standard phase-encoding techniques (4.5 mm thickness and 3.75-by-3.75 mm in-plane voxel resolution, inter-slice distance 1mm, TR = 1600 msec, flip angle = 90°, field-of-view = 240×240×256 mm, interleaved slice acquisition, matrix size = 64×64; 16 slices oriented along the calcarine sulcus) with the modification that two wedges of an 8Hz flicker black and white polar checkerboard grating were bilaterally opposite like a bowtie, to enhance signal to noise [48], [49]. Wedges occupied a given location for 2 TRs (3.2 seconds) before moving to the adjacent location in a clockwise fashion. Each wedge subtended 18 degrees of 360 degrees. 9.6 seconds (6 TRs of dummy scans) were discarded before each run to bring spins to baseline. 168 volumes were collected on each run. A minimum of 7 wedge runs were collected for each subject and then averaged to minimize noise before retinotopic data analysis in BV 4.9.6. A minimum of three runs was collected per subject using expanding 8Hz flickering concentric rings that each spanned approximately one degree of visual angle in ring width. Each ring was updated after one TR (1.6s) after which it was replaced by its outward neighbor, except that the outermost ring was replaced by the innermost ring, whereupon the cycle was repeated. Retinotopic areas (V1d, V1v, V2d, V2v, V3d, V3v, V4v/VO1, and V3A/B) were defined as masks on the basis of standard criteria [48], assuming a contralateral quadrant representation for V1d, V1v, V2d, V2v, V3d, and V3v, and a contralateral hemifield representation for V4v/VO, and V3A/B [50]. V4v and the hemifield representation just anterior to it, called VO [51], were combined into a common mask because the border between these regions was not distinct in all subjects, as was true for the combination of V3A and V3B into a common V3A/B mask (**Figure 4**).

### hMT+ masks

The human analog of macaque motion processing area MT has been called V5 or human hMT+. Left and right hMT+ were localized in 9 of the 14 subjects using a localizer scan comprised of three to six runs of three minutes each. The hMT+ localizer stimuli consisted of a grid of 3×3 subgrids of solid white squares on a black background whose length and height were approximately one degree by one degree. This was constructed by eliminating the zeroth, and ±fourth, and ±eighth rows and columns from a regular grid of squares. Square centers were separated by approximately three degrees. In baseline blocks the grid remained stationary for a twenty-second epoch, followed by an epoch where the grid rotated clockwise around its center at a speed of 270 degrees per second. Each run contained nine epochs of alternating motion and non-motion stimulation. As in the main experiment, subjects carried out a simple fixation task in order to assess visual fixation and wakefulness, pressing a button in their right hand any time the fixation point changed color. hMT+ was localized as activity in the motion > non-motion GLM contrast that survived the threshold p<0.0001 corrected (fixed effects). In addition, activation had to occupy the inferior occipital gyrus or inferior temporal sulcus in order to be localized as hMT+. The mean Talairach coordinates of hMT+ in the right hemisphere were x = 44.7, y = −66.8, and z = 1.5, and in the left hemisphere: x = −40.7, y = −69.9, z = 3.3. The hMT+ masks thus localized could contain area MST as well.

### fMRI Data analysis

Event related time-course averages were constructed on a subject-by-subject basis by averaging within individually mapped retinotopic ROIs, including V1v, V1d, V2v, V2d, V3v, V3d, V3A/B, and V4v. The average timecourse for each of the two perceptual states was constructed by identifying the TR in which a perceptual switch occurred and the 6 TRs that immediately followed the switch. These intervals were categorized into the two perceptual states. The BOLD signal in each interval was normalized so that the value of the BOLD signal at TR_0_ = 0. The normalized BOLD intervals were then averaged together by category.

Because our goal was to compare the BOLD signal that occurred upon perceptual switches, we only averaged data from ‘see’ conditions that followed ‘no see’ conditions, and vice versa. Thus the first button-press in each stimulation block, which always corresponded to a ‘see’ state (induced by the stimulus onset at the beginning of each stimulation block at the end of a ‘no dot’ epoch), was excluded to eliminate nonspecific onset effects that possibly had nothing to do with the reappearance of the vanished dot after a ‘no see’ state. Also, if the last button-press happened within 3 TRs before the end of a stimulation block, it was excluded to eliminate any possible offset effects that likely had nothing to do with MIB. A potential problem with timecourse averaging such as that used here is that variable durations of perceptual states tend to blur the later part of the timecourse of individual responses. In order to avoid this possible contamination, we excluded those percepts shorter than 2TRs. The time-course averaging was carried out for all voxels in retinotopic cortex. Within each retinotopic ROI, the average time-course data was then averaged across all voxels. This resulted in an average time-course waveform for each retinotopic area for each subject. These waveforms were then averaged across subjects.

After excluding those percepts shorter than 2TRs, numbers in the following parentheses indicate the percentage of time that a subject was in the ‘see’ state at a given TR around a perceptual switch to the ‘see’ state: TR = −3 (0%); TR = −2 (0%); TR = −1 (0%); TR = 1 (100%); TR = 2 (100%); TR = 3 (100%); TR = 4 (82%); TR = 5 (62%). Similarly, numbers in parentheses below indicate the percentage of time that a subject was in the ‘no see’ state at a given TR around a perceptual switch to the ‘no see’ state: TR = −3 (0%); TR = −2 (0%); TR = −1 (0%); TR = 1 (100%); TR = 2 (100%); TR = 3 (100%); TR = 4 (83%); TR = 5 (55%). Because of the hemodynamic response lag, we present the timecourse data up to TR = 6, which is presumably indicative of the neuronal processing underlying the conscious state experienced at TR = 4, during which the subjects are in a given state over 80% of the time. ‘See’ states do not include the duration at the beginning of a run, which was always a ‘see’ state, because this state was not a transition from a ‘no see’ state.

In areas V1v, V1d, V2v, V2d, V3v, and V3d, we further show the timecourse for the following three conditions, based on whether the target dot was presented inside (labeled “contra within”), outside (labeled “contra outside”), or ipsilaterally (labeled “ipsi”) to a ROI's corresponding quadrant visual field. In areas V3A, V4v, and hMT+, we only compared the timecourse based on whether the target dot was presented contralaterally (the “contra” condition) or ipsilaterally (the “ipsi” condition) to a ROI because receptive fields within these areas are known to be large, and thus may cross the horizontal meridian if not the vertical meridian.

## References

[1] Bonneh YS, Cooperman A, Sagi D (2001). Motion-induced blindness in normal observers.. Nature.

[2] Graf EW, Adams WJ, Lages M (2002). Modulating motion-induced blindness with depth ordering and surface completion.. Vision Res.

[3] Hsu LC, Yeh SL, Kramer P (2004). Linking motion-induced blindness to perceptual filling-in.. Vision Res.

[4] Troxler D, Himly K, Schmidt JA (1804). Über das Verschwinden gegebener Gegenstände innerhalb unsers Gesichtskreises,. *Ophthalmologisches Bibliothek*.

[5] Ramachanndran V (1992). Blindspot.. Sci Am.

[6] Clarke FJ, Belcher SJ (1962). On the localization of Troxler's effect in the visual pathway.. Vision Res.

[7] Kotulak JC, Schor CN (1986). The accommodative response to subthreshold blur and to perceptual fading during the Troxler phenomenon.. Perception.

[8] Millodot M (1967). Extra foveal variations of the phenomenon of Troxler.. Psychologie Francaise.

[9] Safran AB, Landis TL (1998). The vanishing of the sun: A manifestation of cortical plasticity.. Survey of ophthalmology.

[10] Zur D, Ullman S (2003). Filling-in of retinal scotomas.. Vision Res.

[11] Funk AP, Pettigrew JD (2003). Does interhemispheric competition mediate motion-induced blindness? A transcranial magnetic stimulation study.. Perception.

[12] Mitroff SR, Scholl BJ (2004). Seeing the disappearance of unseen objects.. Perception.

[13] Mitroff SR, Scholl BJ (2005). Forming and updating object representations without awareness: evidence from motion-induced blindness.. Vision Res.

[14] Martinez-Conde S, Macknik SL, Troncoso XG, Dyar TA (2006). Microsaccades counteract visual fading during fixation.. Neuron.

[15] Tong F (2003). Primary visual cortex and visual awareness.. Nature Rev Neurosci.

[16] Castelo-Branco M, Formisano E, Backes W, Zanella F, Neuenschwander S (2002). Activity patterns in human motion-sensitive areas depend on the interpretation of global motion.. PNAS.

[17] Goebel R, Khorram-Sefat D, Muckli L, Hacker H, Singer W (1998). The constructive nature of vision: direct evidence from functional magnetic resonance imaging studies of apparent motion and motion imagery.. European Journal of Neuroscience.

[18] Liu T, Slotnick SD, Tantis S (2004). Human MT+ mediates perceptual filling-in during apparent motion.. Neuroimage.

[19] Muckli L, Kriegeskorte N, Lanfermann H, Zanella FE, Singer W (2002). Apparent Motion: Event-Related Functional Magnetic Resonance Imaging of Perceptual Switches and States.. Journal of Neuroscience.

[20] Muckli L, Kohler A, Kriegeskorte N, Singer W (2005). Primary visual cortex activity along the apparent-motion trace reflects illusory perception.. PloS Biology.

[21] Engbert R, Kliegl R (2003). Microsaccades uncover the orientation of covert attention.. Vision Res.

[22] Martinez-Conde S, Macknik SL, Troncoso XG, Dyar TA (2006). Microsaccades counteract visual fading during fixation.. Neuron.

[23] Tse PU, Sheinberg DL, Logothetis NK (2004). The distribution of microsaccade directions need not reveal the location of attention.. Psychol Sci.

[24] Horwitz GD, Albright TD (2003). Short-latency fixational saccades induced by luminance increments.. J Neurophysiol.

[25] Gawne TJ, Martin JM (2002). Responses of primate visual cortical neurons to stimuli presented by flash, saccade, blink, and external darkening.. J Neurophysiol.

[26] Leopold DA, Logothetis NK (1998). Microsaccades differentially modulate neural activity in the striate and extrastriate visual cortex.. Exp Brain Res.

[27] Martinez-Conde S, Macknik SL, Hubel DH (2000). Microsaccadic eye movements and firing of single cells in the striate cortex of macaque monkeys.. Nature Neurosci.

[28] Sylvester R, Haynes JD, Rees G (2005). Saccades differentially modulate human LGN and V1 responses in the presence and absence of visual stimulation.. Curr Biol.

[29] Tse PU, Baumgartner FJ, Greenlee MW (submitted). Cortical activity evoked by microsaccades, visually-guided saccades, and eyeblinks in human visual cortex.

[30] Brefczynski JA, DeYoe EA (1999). A physiological correlate of the spotlight_ of visual attention.. Nat. Neurosci.

[31] Chawla D, Rees G, Friston KJ (1999). The physiological basis of attentional modulation in extrastriate visual areas.. Nat. Neurosci.

[32] Gandhi S, Heeger D, Boynton G (1999). Spatial attention affects brain activity in human primary visual cortex.. Proc. Natl. Acad. Sci. U. S. A..

[33] Kastner S, Pinsk MA, DeWeerd P, Desimone R, Ungerleider LG (1999). Increased activity in human visual cortex during directed attention in the absence of visual stimulation.. Neuron.

[34] Martinez A, AnlloVento L, Sereno MI, Frank LR, Buxton RB (1999). Involvement of striate and extrastriate visual cortical areas in spatial attention.. Nat. Neurosci.

[35] Müller NG, Bartelt OA, Donner TH, Villringer A, Brandt SA (2003). A physiological correlate of the “Zoom Lens” of visual attention.. J. Neurosci.

[36] Shulman GL, d'Avossa G, Tansy AP, Corbetta M (2002). Two attentional processes in the parietal lobe.. Cereb. Cortex.

[37] Slotnick SD, Schwarzbach J, Yantis S (2003). Attentional inhibition of visual processing in human striate and extrastriate cortex.. Neuroimage.

[38] Somers D, Dale A, Seiffert A, Tootell R (1999). Functional MRI reveals spatially specific attentional modulation in human primary viosual cortex.. Proc. Natl. Acad. Sci. U. S. A..

[39] Watanabe T, Harner AM, Miyauchi S, Sasaki Y, Nielsen M (1998). Task-dependent influences of attention on the activation of human primary visual cortex.. Proc. Natl. Acad. Sci. U. S. A..

[40] Hochstein S, Ahissar M (2002). View from the top: hierarchies and reverse hierarchies in the visual system.. Neuron.

[41] Lamme VA, Roelfsema PR (2000). The distinct modes of vision offered by feedforward and recurrent processing.. Trends Neurosci.

[42] Pollen DA (2003). Explicit neural representations, recursive neural networks and conscious visual perception.. Cereb Cortex.

[43] Super H, Spekreijse H, Lamme VA (2001). Two distinct modes of sensory processing observed in monkey primary visual cortex (V1).. Nature Neurosci.

[44] Crick F, Koch C (1995). Are we aware of neural activity in primary visual cortex?. Nature.

[45] Rees G, Krieman G, Koch C (2002). Neural correlates of consciousness in humans.. Nature Rev Neurosci.

[46] Watanabe T, Cavanagh P (1991). Texture and motion spreading, the aperture problem, and transparency.. Perception and Psychophysics.

[47] Kingstone A, Fendrich R, Wessinger CM, Reuter-Lorenz PA (1995). Are microsaccades responsible for the gap effect?. Percept Psychophys..

[48] Sereno MI, Dale AM, Reppas JB, Kwong KK, Belliveau JW (1995). Borders of multiple visual areas in humans revealed by functional magnetic resonance imaging.. Science.

[49] Slotnick SD, Yantis S (2003). Efficient acquisition of human retinotopic maps.. Hum Brain Mapp.

[50] Tootell RB, Mendola JD, Hadjikhani NK, Ledden PJ, Liu AK (1997). Functional analysis of V3A and related areas in human visual cortex.. J Neurosci.

[51] Brewer AA, Liu J, Wade AR, Wandell BA (2004). Human ventral occipitotemporal cortex contains several visual field maps with differential stimulus selectivity.. Society for Neuroscience Meeting abstract 300.23.

